# Mind-body skills groups for medical students: reducing stress, enhancing commitment, and promoting patient-centered care

**DOI:** 10.1186/1472-6920-14-198

**Published:** 2014-09-22

**Authors:** James S Gordon

**Affiliations:** The Center for Mind-Body Medicine, 5225 Connecticut Avenue Suite 414, Washington, DC 20015 USA; Georgetown University School of Medicine, 3900 Reservoir Rd NW, Washington, DC 20007 USA; The Center for Mind-Body Medicine and Georgetown University Medical School, Washington, DC USA

**Keywords:** Mind-body medicine, Mind-Body Skills Groups, Medical student stress, Self-care, Support group, Group intervention, Patient-centered, Medical education, Mindfulness, Center for Mind-Body Medicine, James S. Gordon

## Abstract

**Background:**

For several decades, psychological stress has been observed to be a significant challenge for medical students. The techniques and approach of mind-body medicine and group support have repeatedly demonstrated their effectiveness at reducing stress and improving the quality of the education experience.

**Discussion:**

Mind-Body Skills Groups provide medical students with practical instruction in and scientific evidence for a variety of techniques that reduce stress, promote self-awareness and self-expression, facilitate imaginative solutions to personal and professional problems, foster mutual understanding among students, and enhance confidence in and optimism about future medical practice. The Center for Mind-Body Medicine, which developed this model 20 years ago, has trained medical school faculty who offer these supportive small groups to students at more than 15 US medical schools. This paper describes the model, surveys its use in medical schools, summarizes published research on it, and discusses obstacles to successful implementation as well as its benefits.

**Summary:**

Mind-Body Skills groups have demonstrated their effectiveness on reducing stress in medical students; in enhancing the students’ experience of medical education; and in helping them look forward more confidently and hopefully to becoming physicians. The experience of these 15 institutions may encourage other medical schools to include mind-body skills groups in their curricula.

## Background

Stress is a hazard of medical training and can inhibit learning and diminish performance. Numerous studies reveal that medical students experience a significantly increased incidence of the psychological, physical, and behavioral disorders to which chronic stress contributes including depression [1, 2], anxiety, and burn-out [3–7]. Others have linked stress levels of medical students to the quality of care they give their patients [8, 9].

Already in the 1960s, several medical schools and hospitals were developing strategies to teach medical students, residents, and physicians to better handle stress and its psychological and physical consequences. Small groups modeled on “sensitivity” or “training groups” which had previously been used with psychiatric patients and mental health professionals, as well as in the corporate world, were implemented [10, 11]. These interventions emphasized group support [12, 13], self-awareness [14], and sharing of feelings [10]. They included discussions on such topics as the physician’s role and its meaning [15, 14] and being female in medical school [11], and provided didactic instruction about group dynamics [11]. Participants experienced greater awareness of their own emotional issues as students, less personal isolation, more sensitivity in communicating with classmates [10], and improved faculty/student communication [14].

During the late ‘70s and ‘80s there was a shift in emphasis from “sensitivity training” to “stress reduction” [16–22] and a deepening understanding of the importance of instruction that promoted self-care. The focus on stress reduction was informed by the accumulating scientific evidence on how physiology may be affected by techniques which use the mind [23] including meditation [24–26], biofeedback [27–29], and guided imagery [30, 31]. This approach was already being shown to be a valuable part of comprehensive care for chronic illness.

In medical school settings the emphasis was on teaching a specific technique: meditation (such as South Asian vipassana or mindfulness meditation [32, 33], self-hypnosis [34, 35], or relaxation techniques including progressive muscular relaxation and deep breathing [20–22]. These programs were taught in both classroom and small group settings.

Studies on medical students who practiced such “mind-body” techniques in a small group or classroom setting demonstrated enhancement of a variety of health indices [36, 33], decreased levels of depression [35, 32, 37], improved immunological functioning [35, 38], and greater general health [18, 20, 39]. Medical students, who benefited from mind-body approaches, in turn, began to realize the importance of these approaches for present and future patients. More generally, they came to appreciate the vital role of self-care (which also includes nutrition and exercise) in preventing and treating chronic illness [39].

## Discussion

### The Mind-Body Skills Group model

The author and his colleagues at the Washington DC-based Center for Mind-Body Medicine (CMBM) developed the Mind-Body Skills Group model in the early 1990’s and began to offer it to people with cancer and other life-threatening illness and to inner-city teenagers and their counselors. In 1993, CMBM began to train health and mental health professionals in Washington DC and later nationally and internationally. Currently, some 5,000 have been trained in and many are implementing the model in hospitals, clinics, community-based organizations, and in primary and secondary as well as professional schools in Bosnia, Kosovo, Macedonia, Israel, Gaza, and Haiti, as well as throughout the US. Published studies [40–42] on war-traumatized populations using the Mind-Body Skills Group model, including the first randomized controlled trial of any intervention for war-traumatized children, have repeatedly demonstrated an 80% reduction in post-traumatic stress disorder and highly significant decreases in depression and feelings of hopelessness. These gains were largely maintained at three and seven months follow-up (in Kosovo and Gaza) and in the case of Gaza, in spite of ongoing armed conflict and 70% unemployment.

The Mind-Body Skills Groups, which ordinarily include one or two leaders and 10 participants, teach a variety of specific mind-body skills including relaxation techniques, deep-breathing, biofeedback and autogenic training, and guided imagery. Several forms of meditation including active (fast deep breathing and “shaking and dancing”) as well as “concentrative” and “mindfulness” meditations are used. Techniques of self-expression in words, drawings, and movement are included. Genograms (family trees) are constructed and shared to highlight strengths and sources of family support as well as physical, behavioral, and cultural challenges that family members have had to meet.

Groups are generally held for 2 hours, once a week, for 8–12 weeks, though shorter sequences are sometimes offered. In the first group, the ground rules are explained. These include: confidentiality; refraining from interrupting others or interpreting or analyzing what they say or do; the role of the leader as both teacher and participant (leaders do exercises along with participants and take their turn in the check-in process); the option of “passing” if one does not want to speak; and the importance of attending all groups and arriving on time or informing the instructor of extenuating circumstances. Group members understand that they are expected to attend, barring emergency, every group. The emphasis is on being “present”, or aware of one’s own thoughts, feelings, and sensations and bringing the same attentive presence to the shared words and experience of other group members.

Each group has a similar structure. It begins with a quiet meditation, often slow deep breathing, in through the nose and out through the mouth, with the belly relaxed or “soft”.

A “check-in” follows in which students and the faculty leader(s) share their experience of the previous week, including their use of techniques they have previously learned and the benefits and challenges they have faced in practicing these techniques. Each group member is also instructed to say how he or she is feeling “right now”. The faculty leader ensures that group members speak in order and without interruption.

In each group the leader teaches a new mind-body technique or form of self-expression (eg. guided mental imagery; a written dialogue with a symptom, problem, or issue; an experience of “mindful walking” or “mindful eating”). The group leader gives a brief talk which outlines the scientific evidence for the approach—for example, presenting recent research on the effects of meditation on brain physiology and structure [43–46]—then teaches it to the students, ensuring that all understand how it’s done. These are presented as “experiments”, opportunities for learning about oneself and one’s experience, and/or as ways for using the imagination and self-expression to identify troubling problems and find solutions to them.

Following the experiential exercise, participants share what they have done or learned: they show their drawings, read written dialogues, or describe meditative or imaginative experiences.

The group concludes with a brief meditation.

The Mind-Body Skills Groups (MBSG) are grounded in a meditative approach and philosophy. Participants are taught to attend to their own thoughts, feelings, and sensations as they do the experiential exercises and as others share. Leaders teach the exercises and facilitate sharing but do not analyze or interpret the student’s responses and ensure that students do not interrupt one another or “give advice”.

The groups give students the opportunity to learn the fundamental principles and the basic science of mind-body approaches, experience their psychophysiological benefits, express themselves freely in a safe environment, develop self-awareness and an intuitive understanding of the origins and solutions to their own stress, and share their experiences of this approach with one another in an intimate and unforced way. Teaching a variety of techniques enables students to appreciate different forms of self-care; to discover which techniques are most appealing and/or challenging to them; and to develop comprehensive and individualized programs of self-care. The focus throughout is on students learning to understand and care for themselves, and on each student’s experience and sharing as a mirror and a source of learning for all others.

A typical sequence of ten groups is:

**Ten Session Mind-Body Skills Group Outline**

**GROUP #1: Drawings***“Yourself”, “Yourself with your biggest problem”, “Yourself with your biggest problem solved”*

**GROUP #2: Autogenic Training & Biofeedback**

**GROUP # 3: Meditation – Active Meditation (“Shaking and Dancing”)**

**GROUP #4: Guided Imagery***“Safe Place” and “Wise Guide” Imagery*

**GROUP #5: Written Dialogue with a Symptom/Problem/Issue**

**GROUP #6: Genograms**

**GROUP #7: Genograms (continued)**

**GROUP #8: Relationship with Food & Mindful Eating**

**GROUP #9: A Second Set of Drawings***“Yourself”, “How you would like to be (personally, and/or professionally)”, “How you will get there”***Mindful Walking**

**GROUP #10: Closing Group Ritual**

In the mid 1990’s the author began using this group model in electives for medical students in family medicine and psychiatry at The Georgetown University School of Medicine as well as in extracurricular groups for Georgetown students who identified themselves as particularly “stressed-out”.

The intention was twofold: one, to give medical students the tools and the support they needed to reduce the high levels of stress, burnout, and sleeplessness that were so prevalent among them. And two, to give them a first-hand experience of the power—and challenges—of self-care so that they, in turn, would be more inclined to include self-care, and, more generally, health promotion and wellness, in their future clinical work.

This paper explores the benefits—and challenges—of implementing MBSGs in US medical schools.

## Methods

From 2010 through 2012, research assistants at CMBM (T.E. and L.E.) as well as the author interviewed CMBM trained faculty from eighteen U.S. medical schools. Fifteen of these faculty had informed CMBM that they had set up Mind-Body Skills Groups specifically for medical students at U.S. medical schools (two others were beginning the process). These faculty were questioned about the format and content of the group sessions as well as challenges in implementation and reported student benefit. Responses were recorded and programs were summarized based on the faculty’s reporting. All participants who are involved in research studies gave written and informed consent. The informants in the survey—none of whom was revealing clinical information, or identifying individual participants in the program--gave oral consent and their responses were reviewed with them.

**Medical School Faculty Mind-Body Skills Group Questionnaire**

1. I understand that Mind-Body Medicine courses are taught at your institution. To whom are you offering these courses? Students only? What year or years medical students?
  Interns? Residents? Are any courses offered to the faculty? Have any additional faculty been trained or will they be trained to teach mind-body medicine? About how many?
2. How is Mind-Body Medicine taught? Is it incorporated into the curriculum as a required course? As an elective course? Or is it taught outside of the curriculum (explain)?
3. In your best estimate, how many students (or interns or residents or faculty, based on previous answers) have taken your Mind-Body Skills course(s)?
  How many groups? How many students in each group? How many weeks? What’s the format of the course?
4. Is the Center for Mind-Body Medicine’s small group model used in teaching these courses? (If not, find out how they are teaching mind-body medicine)
  Which of the modalities of the CMBM training course are being taught?
   Biofeedback
   Guided Imagery
   Autogenics
   Meditation
   Genograms
   Drawings
   Journaling
   Movement (including exercise, yoga, martial arts, and dance)
     What has been added and/or deleted? If not using the Center’s modalities, what were the reasons for eliminating modalities or changing the model if it was changed?
5. Were Mind-Body Medicine courses taught at your institution PRIOR to you attending the CMBM training? (this is fine as a yes/no) Regardless of the answer ask: How long have the courses been taught (inquire about previous instruction and CMBM-style instruction)?
6. On a scale of 1-5 with 1 being “not at all” and 5 being “extremely well”, how well does it appear that students have learned and internalized the training in Mind-Body Medicine? What kind of quantitative or qualitative measures have you used? Exams?
7. In your opinion, how has teaching Mind-Body Skills to medical students changed or affected the quality of their education? Have you seen any personal benefit to the students? If so, please explain. Do you have any research measuring these benefits? Please share. Would you be willing to share your results (or a summary of research)?
8. How did you go about introducing Mind-Body Medicine courses in the institution? Did you face any challenges? Please explain.
9. Have you seen any change or impact on the faculty or climate of the institution as a result of teaching Mind-Body Medicine? Please explain.
10. How have the CMBM training programs affected you personally? Professionally?

The vast majority of these faculty had completed CMBM’s 5-day Professional Training Program, the 5-day Advanced Training Program, 36 hours of practicum work, and had returned to the 5-day Advanced Training Program, after completing three papers on mind-body medicine. Other certified MBSG practitioners may be leading groups at medical schools which were not included in this article. And at some schools, those who have been trained by CMBM have in turn been training and supervising other faculty.

Georgetown and the University of Washington have developed the most comprehensive programs and are described in some detail, as is research on these programs published in peer-reviewed journals. Programs in 13 other US medical schools and the lessons learned from them are summarized.

## Results

### Georgetown University

Georgetown, the original site for MBSGs, continues to have the most robust program. In the 2011 spring semester, 12 groups were running simultaneously with 30% of the students in the first year class participating and 40% of the class taking part in an 11-week long MBSG by the time they graduate. Georgetown has also developed levels 2, 3, and 4 groups that provide ongoing experiential learning and support. Research on the Georgetown program has been both qualitative and quantitative.

Saunders et al. [47] demonstrated that participation in the group enhanced the students experience of “connection”, “self-discovery”, “learning”, “stress-relief”, and “medical education”. Many students reported that the group was a unique and uniquely valuable experience, giving them skills which enhanced their academic performance, helped them to sleep and concentrate more effectively, and enabled them to overcome feelings of isolation that had inhibited and distressed them. The groups taught them to be more “open-minded” about new perspectives and practices, including self-care and a “holistic” approach to medicine and patient care. Students reported feeling cared for by the teachers who led the groups and the students who participated with them.

A study by MacLaughlin et. al [36] provided biological evidence for the stress reducing capacity of the Georgetown groups. Students who participated in the groups did not have the expected increase in salivary cortisol (or lower levels of DHEA-S) at exam time which controls exhibited. There was no difference at baseline between the controls and those in the Mind-Body Skills Groups. An incidental but important finding of the study was that attendance at these groups was 98%.

Because of the success of the program, it has received considerable support from the Georgetown administration, including the appointment of a half-time program coordinator, a clinical social worker, who formerly led the MBSG program at The Center for Mind-Body Medicine. Eighteen faculty were trained by CMBM and an additional 22 have been trained by the program coordinator and other CMBM trained faculty. MBSG group leaders include both clinicians and basic science faculty.

### The University of Washington

The University of Washington (UW) offers six Mind-Body Skills Groups each year for second-year students, a total of 60 students each year. In a 2007 study [48], Finkelstein and her colleagues compared students who participated in MBSGs with a “comparison group of non-enrolled classmates”. Students who participated in MBSGs had higher initial anxiety scores than those in the comparison group. By the end of the course, their anxiety had declined significantly and their levels were “indistinguishable from non-enrolled counterparts”. These improvements were sustained at 3-months follow-up. Though the groups were not randomized, the comparison reflects the nature of groups in many schools: enrollment in these electives is likely to be higher among those who are experiencing more stress.

The UW program has been coordinated and sustained by volunteer faculty. All UW faculty leaders were trained by The Center for Mind-Body Medicine; and here, as at Georgetown, both clinical faculty and basic scientists lead MBSGs.

### Other medical schools

In addition to Georgetown and the University of Washington, at least 13 other medicals schools have offered MBSGs to their students [*see Full List of Medical Schools*].

**Full List of Medical Schools offering Mind-Body Skills Groups**

-  Arizona Health Sciences Center
-  University of Connecticut
-  Duke University
-  Johns Hopkins University
-  University of Illinois
-  University of Kentucky
-  Louisiana State University
-  University of Michigan
-  University of Minnesota
-  Oregon Health Science University
-  Stanford University
-  University of Texas
-  Tulane University

There is considerable variability in the length of the program—from 4 to 12 weeks—with most offering the 2-hour groups for 8–10 weeks. In all but 2 instances, MBSGs were an elective for-credit course with 2 programs presenting the group as a non-credit experience. Some of the electives originated as segments of required courses or were originally offered without credit.

Some of these courses, for example at the Universities of Connecticut and Indiana and Stanford, were presented in ways that were virtually identical to the CMBM model which Georgetown and the University of Washington have adopted. Others, including the University of Arizona, Duke, and the University of Texas, have included elements of other programs (including Mindfulness-Based Stress Reduction and The Healer’s Art) but have generally retained the structure and spirit of the Mind-Body Skills Groups. All appreciate a model which is flexible enough to include a variety of approaches to self-awareness, self-expression, and self-care.

According to the interviews with program leaders, none of these other schools have published research on their program. In structured interviews, however, program leaders describe benefits that were quite similar to those revealed in formal research at Georgetown and the University of Washington. These included:

-  The importance of having a “safe place” where students could share thoughts and feelings without fear of censure or judgment. Students often said that these groups were the “only place where I could be myself”.
-  Self-reported reduction in physical and psychological symptoms of anxiety, depression, insomnia, and headache.
-  Students, all but unanimously, felt less competitive with and more understanding of and compassionate toward one another.
-  Students felt a “recommitment” to medicine, to patients and their future as physicians and to a “life of service”.
-  Students gained an understanding of the crucial importance of self-care for themselves and for the health of their patients.

Faculty also spoke of their own satisfaction in leading these groups and practicing the techniques they taught the students. Like the students, they felt that participation in the groups lowered their levels of stress; enhanced their openness to more “humanistic”, “patient-centered”, and “innovative” approaches to healthcare; and encouraged them to appreciate the importance of self-care for their patients as well as themselves. They felt, as well, a greater, more personal connection to the students in their groups and took satisfaction from helping them to embrace their profession more whole heartedly and with a deeper commitment to their patients’ welfare.

Challenges included:

-  In many cases, faculty lead these groups as volunteers in addition to all their other responsibilities. Only at Georgetown, is there a faculty member whose time is specifically underwritten to coordinate and supervise the program. Administrators, though recognizing the benefit of MBSGs to students and encouraging enrollment in them, have not, except in the case of Georgetown, set aside funds for coordinating and leading the programs.
-  Many of the most robust programs, including those at Georgetown, the Universities of Washington, Connecticut, Michigan, and The Oregon State Health Sciences University were begun or greatly enhanced with the support of NIH R25 grants for “Complementary and Alternative Medical Education”. After these grants expired, there was no longer funding for coordinating, supervising, and implementing programs so that these responsibilities were taken on by committed volunteers.
-  Faculty often face limited time for electives in students’ schedules, particularly in the first two years when students are generally most enthusiastic about participating in MBSGs.
-  Competition with other elective and non-credit courses which also appealed to students’ concern with reducing stress and increasing personal awareness and their role as patient-sensitive physicians limited enrollment in some institutions. Most often cited were classes in Mindfulness-Based Stress Reduction and The Healer’s Art.

### Further discussion

Mind-Body Skills Groups have been offered to students in more than 15 US medical schools. They are likely, as faculty continue to be trained by the Center for Mind-Body Medicine, to be offered elsewhere. Both published research, and semi-structured interviews with course directors reveal decreased levels of stress (improved mood and symptoms of stress-related conditions), greater resilience, better performance, enhanced connectivity among students and between students and faculty group leaders. The groups helped students look forward more hopefully to their roles as physicians committed to a holistic and compassionate patient-centered care. Having themselves experienced the benefits of self-care, these students (and their faculty group leaders) now believe that teaching self-care will be an important part of their work as physicians and their interactions with future patients.

The success of these programs appears to depend on several sometimes related and mutually reinforcing factors including: the position, influence, and commitment of MBSG’s champions; the number of faculty trained; the presence of financial support for the program; the flexibility of the medical students’ schedules; and the ongoing connection of MBSG leaders to The Center for Mind-Body Medicine.

Where the program’s original champions held important positions—as department chairs, deans, or influential members of the faculty, the programs have generally served more students and continued for many years, undiminished in scope and appeal. This has been true at Georgetown, the Universities of Washington, Connecticut, Indiana, and Oregon Health Sciences University. In a number of cases, these influential faculty significantly expanded or, indeed, launched the MBSG program with the aid of NIH R25 funds.

The number of faculty trained is also an important factor in the sustainability of the program and is in some cases related to the original R25 funding. Georgetown, where 18 faculty participated in the Center for Mind-Body Medicine trainings, and 22 more have subsequently been trained by Georgetown’s leaders, is by far the most extensive MBSG program, with The University of Washington, The University of Indiana, and The University of Connecticut next. The Georgetown program has benefitted significantly from the presence of a half-time paid coordinator who organizes all aspects of the program and provides ongoing regular supervision of Georgetown faculty.

In other schools, where fewer faculty have been trained, the program is smaller and more vulnerable to faculty departures and reassignments, as well as to changes in the curriculum. It appears, however, that students at these schools receive the same benefits from membership in the groups as those in the larger programs.

Faculty commitment and skill are important factors, as well as the model, in eliciting student satisfaction. It would appear, too, that faculty MBSG leaders who maintain strong connections to the Center for Mind-Body Medicine—participating as faculty in CMBM trainings or returning frequently to CMBM events—also feel supported in continuing to lead MBSGs. These include faculty from Stanford, Tulane, Louisiana State University, Kentucky, The University of Indiana and Johns Hopkins.

The MBSG program continues to evolve and, in a number of instances, to grow. A number of faculty (eg. at UW, Johns Hopkins, LSU, and Duke) have offered MBSGs to residents and fellows. Some, for example at UW, have included nursing students, and Stanford’s faculty has welcomed participation from undergraduate pre-meds as well as first and second year students. Others have offered MBSGs in clinical settings for patients with a variety of chronic conditions and for prevention. At Georgetown, the model has now been incorporated at the Business and Law schools. In a number of cases, faculty who have been recently trained are beginning by offering MBSGs to residents, patients, and staff before they offer these groups to medical students. This is the way new programs are now evolving, for example, at Albert Einstein and the University of Tennessee.

## Summary

Medical schools that actively support the formation and evolution of Mind-Body Skills Groups offer students, and ultimately their patients, multiple benefits. MBSGs are an effective way to help medical students to deal with the high level of stress from which they continue to suffer. They give students a first-hand experience of the effectiveness of self-care in improving sleep, decreasing anxiety and enhancing performance, and encourage students to teach the principles and practices of self-care to their patients. The groups provide a safe haven for students in which they can understand and know themselves and other students in a more thoughtful and compassionate way. The MBSG experience appears to reconnect students to the idealism and passion which led them to choose medicine as a profession. Participation in MBSGs is limited by lack of funding and time in the academic schedule.

When greater encouragement and support from medical school administration is present, these groups can become an important, energizing, and deeply satisfying part of the curriculum, an experience which can potentially enrich all aspects of students’ learning and practice. Evidence summarized in this article demonstrating these multiple benefits should encourage schools with MBSG to continue and expand their support of them. For those currently without MBSG, the evidence suggests that for a small investment this offering can enrich all aspects of students’ learning and practice. As medical student interest in, and public awareness of, the importance of mind-body approaches and self-care increases, this curricular innovation is timely and critical.

## Authors’ information

James S. Gordon is Founder and Director of The Center for Mind-Body Medicine in Washington, DC and Clinical Professor in the Departments of Psychiatry and Family Medicine at The Georgetown University School of Medicine in Washington, DC.

## Abbreviations

CMBM: The Center for Mind-Body Medicine
MBSG: Mind-Body Skills Groups.
